# Address, Delivered before the American Society of Dental Surgeons, at the Opening of its Fourth Annual Meeting

**Published:** 1843-09

**Authors:** Chapin A. Harris


THE AMERICAN

### JOURNAL OF DENTAL SCIENCE.

VOL. IV.] SEPTEMBER, 1843. [No. 1.

### ARTICLE I.

*Address,* delivered before the American Society of Dental Surgeons, at the opening of its Fourth Annual Meeting;—Baltimore, July 18th, 1843. By CHAPIN A. HARRIS, M. D., D. D. S., &c. &c. &c.

*Mr. President and Gentlemen*
*of the American Society of Dental Surgeons:—*

THE welcome return of our anniversary meeting, the fourth of this Association, calling together so many talented and influential members of the profession, for the purpose of devising ways and means to advance the interests of an important branch of the curative art as well as the public good, is an occasion of grateful joy and hearty congratulation. From widely separated sections of the country, from our various fields of labour, usefulness and profit, with spared lives and obligations, a thousand-fold increased, of gratitude to the great Supreme Preserver, we meet to promote the great cause of humanity, by strengthening each others' hands and accomplishing ourselves more perfectly in an art and science so intimately connected with the functions of health in the human system, with the enjoyment of life and the perfection of beauty, as are the operations of Dental Surgery in ameliorating the sufferings and increasing the happiness of man. There is a pleasure as well as a grandeur in contemplating these annual re-unions of members of a time-honoured and useful profession, coming together as to a mighty shrine, before which to renew the truthful vows of deep devotion to the great cause of human good,—and upon whose altar to lay the offerings of study, research and experience. In this annual action there is analogy to the arterial and venous circulation of the blood of the human system; thrown from the central ganglion, it rushes through the canals of the arteries, then into the veins, perambulating the extremities of the body, taking in the needful supply of oxygen from contact with the air in the lungs or near the surfaces—then returning to leave its richness in the great treasury of life, to deposite all its acquired energy at the central seat of vitality; so, at stated periods, the members of this Society come from their posts of observation to report progress at the centre, to throw their discoveries, improvements, the results of industry, research and experience into the common fund—the great joint stock of dental knowledge. And, it is a subject of sincere, fervent congratulation that, so far as we have information, no member of this association has, during the past year, been called from this scene of his earthly labours. All yet live to assuage the bitterness of human infirmity, to illustrate the triumphs of art over disease, and to remedy the imperfections of even nature itself.

In all great movements, either in religion, politics, science or benevolence, the American character and the popular genius of all our institutions immediately direct us to lay hold of the great principle of voluntary association, as an agent of stronger moral power than any known in the country, to effect any desirable good. We have no wealthy and powerful privileged orders to command any given amelioration or improvement, and hence we combine the power of popular number and bring it to bear upon the objects we desire to accomplish. This national peculiarity accounts for the numerous benevolent and scientific associations found in the United States, before the moral power of which, vice, ignorance, empiricism, bigotry, oppression, and long established habits and modes of thinking are fleeing away like mists of morning before the rising sun. This will furnish the reason why America can boast of the first society of Dental Surgeons ever associated together in the world; and if this association shall produce results in any manner comparable to those produced by other American associations, now the wonder and praise of the world, the character of an American Dental Surgeon will stand as high on the archives of science as do the names of Washington, Franklin, Hancock, Henry and Jefferson on the roll of freedom, or Mills, Judson, Perkins and Hill in the annals of American missionary benevolence.

The benefits that result from association, as great as they undoubtedly are in commerce, politics or the agricultural interests, are still greater in science—for the plain reason that the results of the gainful and thrift-increasing pursuits furnish incentives to action that the occupations of art and science fail to supply. The acquisition of knowledge is not always the acquisition of wealth, although knowledge always is power. Thus the more striving and bustling pursuits of life have, in general, greater charms for men than the more studious and sedentary, and the student is thus compelled to make many sacrifices of feeling and inclination, especially in the earlier part of his career, while he is accomplishing his mind, maturing his judgment, and arming himself with power to combat disease, or remedy the defects of nature. Science, long pursued, may indeed lead to wealth, but the man who cultivates science merely for the wealth it will give him, can scarce be actuated by that noble enthusiasm of mind on which true perfection in all science is based. It also leads to consideration among mankind.

So necessary is association to the advancement of science, that the latter has rarely flourished without the aid of the former, and that age of the world is found ever the most illustrious in letters and science in which the bonds of scientific association were drawn the closest, and the brotherhood of art the most warmly cherished. In aid of the great professions of life, the various institutions, so famous throughout the world, were at first organized. From small beginnings and dubious experiments, they have risen to gigantic strength, throwing out annually, in their voluminous transactions, the aggregate wisdom of such a mass of mind, acting under the strongest impulse of competition, and the desire of distinction among the wise and learned of the age.

The history of one scientific association is generally the history of all. They come to a maturity in numbers, influence, respectability and power over public opinion, sooner than their most ardent friends anticipate, becoming almost a wonder to themselves, as the history and experience of the Society which I now have the honor to address, fully testify. Three years ago, about fifteen or twenty gentlemen composed the whole of this association, now embodying upwards of a hundred and thirty members, found in nearly every part of the Union and in adjacent islands of the ocean. Such an accession of talent, influence and members was scarcely dreamed of by the most sanguine of its friends; and equally vain will it be to attempt any estimate of the future increase of the Society, or the good that will result from it. Time only can unravel the destinies of this organization, commenced as it was in accordance with the spirit of the age, and urged on by the claims of nature upon the aids of art and science. No one can wonder at such speedy results of concerted action, who has ever reflected on the power of involution, possessed by principles as well as numbers,—a progressive quality of association, strengthening with progress, and gathering weight and volume with the increase of its movement.

The numerous and highly important improvements that have been made both in theoretical and practical dental surgery, in this country, during the last ten years, indicate the stand that American dentistry is expected to take on the broad theatre of the world, amidst the national rivalry of the elder continent, the hoary home of the arts and sciences, when our land was hidden from the race of man by an untravelled ocean and boundless forests.

The highest and most ardent ambition should be cherished by the dental profession in this country, "redeeming the time," and putting each moment to its appropriate use as a part and parcel of the national character for useful science. Ten years ago, an accomplished dentist might have been pardoned for the thought that his art had arrived to perfection; but what astonishing discoveries and improvements have been made in this department of surgery within that time! And who shall say that the succeeding ten years may not be as pregnant with improvements as the past?

One of the most beautiful features in dental surgery is, that, while its operations are confined to one set of organs, its studies and principles must of necessity embrace the whole body; for, long since, has the question "how *soon* can one be qualified for any branch of medicine or surgery?"—been substituted by the inquiry, "how *well* can he be qualified?"—Time spent in study, when compared to knowledge acquired, is but a trifle; and the institution of the American Society of Dental Surgeons recognizes the great principle that each member is still a learner—that he is teachable as well as teacher—and auditor as well as lecturer—a student as well as a professor.

To the keeping of scientific dentistry has been committed the care of the diseases of the mouth, the seat of the sense of taste, from which so great a portion of the happiness of existence is derived. The dentist has to do with organs in whose condition, health, beauty and comfort, are involved—organs that are connected with nerves which throb with the keenest agony in suffering, or thrill, tremulous with the most delightful sensations in the full tide of health and enjoyment; and with organs so vital, planted so close to the brain, the sensorium of the system, skilful should be the hand, steady the purpose, and perfect the knowledge of the artist and operator, whose work lies so near the throne of being.

To the general stock of knowledge which it is the design of the American Society of Dental Surgeons to accumulate, each member should contribute. The learner should assume the station of teacher, and communicate as well as receive. Mere silent membership should content no one. All the mental and physical energies of every member of the association should be summoned to produce something worthy of the public depository of the Society's knowledge; and with a view to this, we should all be continually adding to the stores of our knowledge and experience, and never consider our professional education as completed. As we become more and more accomplished in our profession, we will the more discover the connection of the dental apparatus with the structures, functions, diseases and sympathies of other organs of the body, and thus be constantly acquiring information that will make us more and more competent to the mastery of the difficult and intricate cases that are continually coming up in the practice of every dental surgeon. Let us not then consider our membership in this Society as a signal for relaxation in study, or as a diploma that we have reached the Ultima Thule of professional knowledge. No man can ever reach the farthest boundary of true science; a life time is too brief for the attainment of absolute perfection in art.

Therefore, membership in this Society should be regarded only as entering into new obligations to apply ourselves more diligently to the cultivation of the science and art of dental surgery, and while we are doing this, we will at the same time be elevating the profession we have chosen for life—for usefulness—for fame.

It is a law of the human mind that no fixed standard or bound of perfection can be considered the *ne plus ultra* of achievement. The attainments of industry and perseverance are always in the ascending series, higher and higher, the effort only a short distance behind the conception, and the design ever growing more perfect on the base of the previous achievement—and the work again still more complete in proportion to the improvement of the plan, aided in the execution by the increasing acquisition of experience, and the progress of taste and the creative faculties towards that sublime point of perfection that may be found only among the unchanging models of a perfect creator.

Thus the great masters of statuary in Greece and Rome, each one endeavoured to form a perfect statue of

"The goddess that enchants the world ;"

and although the might of stupendous genius and long years of patient toil were expended in the effort, one single statue could never express all the living sentiments of beauty. Another artist would strike upon another lineament of touching tenderness and emotion, and another upon another, until nature would weary in loving the impress of mind and passion given to the chill and unthinking stone; and still the work has not been done, and modern sculptors are in as earnest chase after the ideal perfection of beauty as were the ancient. Thus should it ever be in that profession which unites art and science and binds them as vassals and ministering spirits to the pleasure, comfort and health of man.

The honor of science is implicated in the transactions, whether worthy or unworthy, of the American Society of Dental Surgeons; and, if any body of men in the country can be considered the life guards of the dental science, it is this national society. To us are committed the interests both of the art and its principles for the continent, and dentistry itself shall be elevated or depressed in the great—the sacred trust. The dignity of the commission we hold includes, in its consideration the elements of national character, and as we execute it, so will our branch of the great scientific character of America stand out in bold relief, or fall back into shade and obloquy. The high renown of the other branches of the profession of surgery and physic, summon us, "trumpet tongued," to illustrate our branch, crown it with respectability, and make its exercise a blessing to humanity, and an honor to the country in which it is practised. We should feel, while we hear almost daily of the splendid achievements of general surgery—the triumphs of skill over disease—as did the ambitious youth of antiquity whose sleep was taken from him by the victories of a successful conquerer; he could not rest while another was reaping such a harvest of glory—neither should any member of this association while surgery and even our own branch of the curative art are performing achievements every day, that a few years since would have been considered as impossible—as beyond the reach of human skill.

The dental as well as the general surgeon, has to deal with living organs—organs connected most intimately by a thousand sympathies with almost every part of the human frame, and liable to be the cause of disease in other parts of the system, as well as to suffer in the derangement of other organs. No organ is more closely allied to the nervous system than the teeth. This, of itself, calls for a thorough and correct knowledge of the nervous economy, for knowledge of every affinity connecting the facial nerves with the dental arch, so as to enable us to manage all those numerous cases of neuralgia, so distressing to be borne, and so hopeless of cure in the hands of ignorance and quackery. The general surgeon indeed, is often called to perform operations of greater magnitude than the dental surgeon, yet his department never calls for any more mechanical or artist-like skill than dentistry does—nor are his operations generally of a more minute and delicate character, than are those of the dentist. The one deals with flesh, muscle and bone—all of which, when wounded, have in themselves a renovating power of granulation and reproduction ; the other deals with organs which have no inherent power of recovery from the ravages of disease, where art must meet and resist the assaults of the destroyer, and where skill alone can repair the infraction of accident, violence or decay. While the former removes the unhealthy or hopelessly diseased limb, the operations of the other must meet disease at its threshold, and pluck from the system the slowly germinating seeds of organic derangement, and as nature has combined utility and beauty in the teeth, they should be watched with the most assiduous attention and consummate skill. Some oriental writer calls the teeth the "pearls of beauty," and it follows that the accomplished dentist would be the "pearl keeper"—the conservator of those organic gems, which, once lost, have no reproductory power, and can only be substituted by the hand of art instructed in the schools of science.

The enthusiasm of an artist in dentistry has full scope—the creative and imitative can come into full play—all is not dry study, theory, abstract principle,—but it also can boast the excitement of the active mechanical arts—and thus the weariness of the student can be refreshed by the alternation of his instruments, the process of successful operation, and the satisfactory sensation of successfully copying nature, remedying her defects, adding ornament to beauty, and giving health to the system. There is, therefore, no excuse for want of enthusiasm in the dental profession. The dentist who does not feel the great impulsive enthusiasm of his work, of his designs, of his operations, will never carve his name on the monuments reared to skill and excellence by a grateful and honor-giving public.

But what are the associated duties we owe to our profession as members of the American Society of Dental Surgeons—what are we to do collectively and individually to bring our profession to the highest perfection of influence, honor and reputation? And if in the answer to these questions, this opening address shall assume the character of utility rather than display—of plain common sense suggestions rather than polished diction and harmoniously rounded periods, it will be better suited both to the speaker and the occasion. All great associations, and even the most magnificent enterprises among men, must be conducted on plain principles and in accordance with simple rules, easily understood and rigidly observed. Thus the foundation principle of all scientific associations and societies, as in national governments, is a surrendery of a few individual rights—the sacrifice of a little individual ease—for a general and a greater good. True generosity has often been called the refinement of selfishness, because of the real gain which ever follows acts of noble and self-denying kindness; so the giving up of private opinion sometimes, and the deference which one individual should ever pay to the opinion and the will of the many, are concessions of private right almost as sure to be repaid ten-fold, as are individual concessions to government to be repaid in protection, power, and general consequence and estimation among mankind. As one surgeon dentist, however skilful, learned and successful he may be, can single-handed and standing alone in society, earn only a reputation and fortune for himself, which, at death cease, and belong to him as a living man no more;—the necessity of associated effort and influence becomes apparent, and the American Society of Dental Surgeons, kept up in peace and concert from generation to generation, shall become like the Persian band of immortals, a living member stepping in to sustain the station of each one who may fall in the exercise of his profession, and ripe with all the virtues of his calling and an extensive usefulness among his fellow men.

The necessity of this association to secure and perpetuate dental reputation is apparent to every well informed dentist. The world at large never can know what an immensity of labor, study, mental and manual discipline it requires to accomplish a man thoroughly for the skilful practice of Dental Surgery, and this Society can only, as its members act collectively, appreciate individual worth in the profession, and if they do this, their archives are destined to become wealthy in the record of departed merit, as well as the rich repository of living research, genius and literature. Already have the muses smiled from Helicon on dental toil, and the goddess Hygeia, blushed with a more rosy beauty at the song of enchantment which celebrated the achievements of the art, and its connection both with health and loveliness; and other poets shall arise, like Darwin, Marmont and Brown, to crown the pale brow of "star-eyed science" with bays of poesy!—

Take care of yourselves; your fame—reputation—your men of talents—your improvements—discoveries—inventions—and every thing relating to your noble art, would be the injunction which the Genius of America, could she make her voice audible to mortal ears, would address to you! And to do this fully and effectually we must, with one accord, enter heartily and energetically into the great and sublime spirit of association—take hold of the mighty strength of numbers, and honor science itself by elevating and sustaining ourselves on that eminence of moral, intellectual and social dignity, where every votary of science should ever be found.

Let it not be thought presumption in your speaker, selected to deliver the opening address, commencing the present labours and sittings of this respected body, to make some plain suggestions in regard to the best manner of sustaining our profession, our society, and ourselves as dental surgeons.

1. Selfishness must be succeeded by a generous and conciliating spirit of self-denial. The christian virtues look lovely and shine every where. If any one member expects to obtain all the consideration, and wear all the honors of the Society, already so large and destined to become still more numerous, learned and influential, I will venture to say he expects too much, and that which can never reasonably be accorded. No man, however long he may have been in the profession, or however eminent he may have become in it, has a right to be considered infallible; nor should he feel chagrin or the bitter gnawings of envy, if some younger man, some one who came later into the field of dental surgery, aided perhaps by a more liberal course of study, or a more elevated structure of mind, should dissent from his theories, and institute a bolder practice on a broader basis of reasoning. Farewell to peace and honor and justice and truth and well-grounded fame, if the demon envy be permitted to take a seat in the halls of science—to hoot, like an owl, when night's sweetest bird of song pours out the tender melodies of sympathy—or curse, like Haman, at the rise and prosperity of others.

2. Too obstinate a tenacity to favourite theories, and too bitter proscription of the theories of others, should be surrendered to preserve the harmony of associated bodies. The fact that there is generally something good in all theories—something that may be winnowed, like wheat from chaff, even from grotesque absurdity, should make us all tender even of each others errors. And to run into erroneous speculations, at times, proves the activity of the mind that thus wanders, and gives promise that such vigorous exertion in search of truth will soon find the priceless pearl, even if it were sought in the wrong direction at first. General and temperate discussions in associated bodies, and an extended and teachable correspondence with eminent practitioners, have the double effect of making and cherishing lasting and valuable friendships, and settling favourite theories on foundations that time, which destroys most things, shall only beautify and consolidate.

3. One of the first principles of an association like this, should be a friendly spirit between the members,—mutual respect for each other, and that condescension and deference which prevail among gentlemen everywhere. The idea that professional men must be Ishmaelites towards each other, and while they build up their own reputations with one hand, carry a sword in the other to repel attacks or assail others, is a false one, and has done more to discredit learned professions than all other causes combined.

If we, brethren of the same profession, cannot use each other well, how can we expect the world will treat us with consideration and respect. There is in the community a stern disposition, whenever a member of a profession indulges in tirades against his professional brethren, in detractions, vituperations or sly inuendoes, to consider the whole profession either as wanting in courtesy, or ignorant pretenders—the one as bad, only a little more abusive than the other. If we as members of the American Society of Dental Surgeons, pursue this suicidal course towards each other, we shall both earn, deserve, and receive our full share of infamy and disgrace, which shall burn the deeper through skin, flesh and bone, inasmuch as a brighter destiny had been entrusted to our keeping, and we had proved ourselves recreant to our interests, and, indeed, unworthy of any better fortunes.

4. Our common safety and that of the community should make us as honorable men, implacable against the advances of quackery. The well-informed dentist can hold no fellowship with ignorant pretension. War is on his shield against the man, who, in this age of the world has not enough of honesty and ambition to qualify himself to perform highly important operations on delicate and most sensitive organs of the human body—organs so highly essential to the enjoyment of life, health and beauty, as are those of the dental arch.

5. The character of our profession and Society imposes on us the sacred duty, by advice, counsel, warning or direct assistance, of aiding those who may have been unfortunate, or who are just setting out in the profession. The very vigor and nerve of our opposition to quackery, should arm us with sympathy and consideration for all who are endeavouring to qualify themselves for respectability and usefulness in the profession. The old and well-learned practitioner will lose nothing by this generous attention to the interests of another; but by it, he binds the stranger and young aspirant to him by the strong cords of gratitude—makes him ever his friend, and convinces him by deeds that cannot lie, of the real existence among professional men of a hearty, noble, generous sympathy in the welfare of another.

6. The duties of our brotherhood in the great cause of science impose on us the obligation, when any matters of difference shall come up, (and come up they probably will,) between any of us and a professional brother, that we should first go to him, and learn his cause of grief, action, or offence, before the matter is given to the four winds, and all the babbling tongues of scandal to twist and torture into the most monstrous shapes of moral obliquity. One such friendly visit oftentimes puts out a flame of rising anger that might have illuminated a wide district of country, and in its lurid glare shown only the dark and diseased shadows of the human heart—the black vortices of shame, revenge, and every ungenerous, unlovely passion which the tempter of man ever planted in the deserts of the soul. A man never shows his weakest and darkest points of character, until he gets into a bitter and most implacable controversy with another; and then, when he is pouring out gall and wormwood upon the naked head of his antagonist, and, as he supposes, using him up, root and branch, before he is aware, he finds the whole public in possession of the fact, destructive forever of his own fame and reputation, that he is a quarrelsome, and unfriendly, and revengeful man; and he finds out too late that his great controversy has been against his own character, and that he has, indeed, come off most irretrievably triumphant! A little patience, forbearance and investigation will set most matters at rest before the winds howl from the wilderness, and the turbid waves of passion begin to roll.

7. For the purpose of securing or obtaining practice, no honourable, high-minded and well educated dentist will perform his professional offices at prices reduced so low as not to afford him the best materials for his operations, and a fair, generous compensation for a proportionate part of the time and expense of his education, and for the time actually spent in performing the operation. He should not be extortionate, and like the dentist in the story, who had but a single case in the year, charge a year's salary for the extraction of a single tooth; but yet he should never undercharge a professional brother for the sake of drawing away his patients. This is an act of meanness which even the want of bread would hardly justify. It is suicidal, for the man who performs cheap operations, by the common sense of mankind, is ever held in cheap repute, and always rated at his own price. It is suspicious to boast of cheap operations. The question comes up always,—can they be good operations? and good operations they cannot be, unless they be executed in the best manner, and with the best materials.

But, before I dismiss this part of my subject, I beg that any remarks I have made may not be construed as an assumption of advice, or as proof that I believe any of the dangers against which I have warned gentlemen of the society, or any unseemly practices derogatory to the dental profession are now in existence. I warn only against what may, in the course of time, become possible—against far distant, and, I would ardently hope, forever remote dangers. The great and high interests of the association; its lofty stand as the first regularly organized society of dental surgeons in the country, if not in the world; the deep solicitude I ever have felt, and must ever feel in its welfare,—all must be my apology if I have pressed any point of etiquette, or any standard of professional perfection too far or too high. I would lay to my own heart the spirit of my cautions with as much earnest severity of faithfulness as I would wish any other member of this Society, where so many may exceed me in professional accomplishments—if not in ardour in the common cause of science.

Among the general topics of interest to the American Society of Dental Surgeons, I doubt not that the Baltimore College of Dental Surgery will be considered of sufficient importance to merit notice on the present occasion. The first College of Dental Surgery ever known in the world, cannot but be an object of interest to this association. The fact that a thorough dental surgical education can be obtained at this institution, and that those who graduate in it, go out with advantages that can seldom be obtained from private instruction, will ever, it is believed, connect its destinies with the welfare of the profession in this country. Those who have graduated in it have gone forth with much of a general surgeon's knowledge of anatomy, with much of a physician's knowledge of physiology, pathology and therapeutics, besides an amount of special surgical knowledge incident to the peculiar branch of their own profession, and a knowledge of the various mechanical manipulations pertaining thereto, that they might have spent years in vain to acquire alone and uninstructed.

The importance and even necessity of greater facilities, than those which have hitherto been enjoyed, for the acquirement of a thorough dental education, is felt, and cannot but be admitted by the members of the profession generally. Few think of entering into the practice of general surgery and medicine, without the advantages of collegiate instruction in those departments of science. Without this necessary preliminary preparation, few become thoroughly qualified for, and gain a footing in respectable practice. And do the practitioners of surgery and medicine need a more careful, finished and thorough training than do those of dental surgery? Every well-educated dentist will answer, No! Shall a profession then that has suffered so severely as has ours from the accession, to the exercise of its duties, of the ignorant and unlearned, if not unprincipled and designing, not endeavour to remedy the evil? The acknowledged fact, so honorable to those concerned, that many have reached a proud professional eminence without college advantages, is no argument that they are not needed. These exceptions to the general rule, the eminent self-made surgeon dentists of the country, all will bear witness how hard, almost impossible, it was to achieve their reputation by their own unassisted energy; and all such, I have no doubt, will have satisfaction in extending to the profession, and to the country, every advantage and facility for the acquirement of a knowledge of the art, by aiding and encouraging all well conducted efforts for the accomplishment of this most desirable and highly important object.

The beneficial effects resulting from the publication of the American Journal and Library of Dental Science upon our own immediate branch of the curative art and the standing of the profession in the community, are such as should commend it to the brotherhood generally. It has become the medium of intercommunication between the members of our profession—the repository of much valuable information—and the arena on which discoveries, principles and modes of practice are discussed and put to the test of experience. In view then of the advantages that result from it, it is to be hoped that it will continue to be sustained and prosper as a rich evidence of the industry, vitality, intellect and energy of the society whose organ it is, to disseminate its principles and sustain its character.

The formation of the Virginia Society of Dental Surgeons since our last meeting, is an event that should be hailed with pleasure by the members of this association, inasmuch as it gives promise that numerous other similar societies will be organized, at least, in the larger states of the Union—the very existence of which will be a chill to empiricism, a nipping frost to the precocious flowers of dental imposture. The formation of the Virginia Society, as perhaps most of the members of this association already know, was the result of a convention, assembled in the city of Richmond, December 12th, 1842; and may we not hope that the example of the "Old Dominion" will be followed by our professional brethren in other states. Indeed, it is in the power of the members of this, the American Society, not only to have state societies auxiliary to this, established in all the states, but also to have state laws passed, guarding the community from the injuries and deceptions that are annually practised upon it by ignorant pretenders to dental knowledge, whose acquaintance with the principles of dental surgery is too limited and superficial to enable them to pass an examination before a body of men competent to judge of the qualifications necessary to be possessed by those who practise in this department of surgery. This legal protection against empiricism would be of vast importance to the welfare of community, inasmuch as the more it would depress ignorant pretension, the more it would elevate real science, and give importance to those whose habits of study and perseverance have made them masters of the profession.

In most countries, and in most of the states of the Union, the laws have instituted guards around the professions of general surgery and medicine, protecting them from the approaches of ignorant pretenders, thus guarding human life, and promoting the public good; but dentistry has been an open field, without any of the salutary enclosures of legislation to protect it from the depredations of any adventurer who might choose to prowl through the community. This is an evil which the profession generally, should endeavor to have remedied. The attention of this association was directed to the subject, at its last anniversary meeting, and a resolution was then adopted, recommending its members to use their exertions to obtain the passage of laws, in the states in which they respectively reside, for the protection of the public against empiricism in this department of the curative art. Indeed, the propriety of the formation of state auxiliary societies, for the purpose of securing the more speedy accomplishment of this object, has been discussed by several of the members of this body; and the subject is certainly worthy of consideration, and from the favourable manner in which it seems to be regarded, I doubt not that it will receive the attention of this association, and be annually recommended, until the escutcheon of dental surgery in the United States, shall boast as many stars as the banner of the Union, and until this body, sustained by twenty-six state societies, shall assume the station in this department of science which the American Congress sustains in the political relations of the country. Then will our resolutions, our discoveries and improvements, and the great impulses we may give to science and art, be taken up, and reverberated from the sunrise to the sunset of American empire. Then shall our acts be re-echoed to the basis of the Rocky Mountains, and through their stupendous gorges, the mountain gates which the Creator opened in the great West, to the far shores of the Pacific Ocean.

There is something beautiful in the contemplation of the introduction and growth of the dental science in the United States. From Europe the first glimmering lights of the profession were introduced almost by chance, like wandering rays from a distant source. But those rays fell upon a country full of the boundless materials of science—a luxuriant field for its development—an acute, enterprising, inventive people—and no wonder that the result has been thc astonishing perfection to which it has now attained. And, while its progress here is flattering to the pride of our country, it is but just to acknowledge that the instructions received from the "father land," constitute the corner stone which lies at the base of the lofty American column of dental surgery.

The first knowledge of the science introduced into our country, about seventy years ago, along with the antagonistic armies of England and France, and the teachings acquired by some American youth from the great masters of the art in France and England, have been the sources of most our subsequent improvements. From these small beginnings the first dental journal, the first association of dental surgeons, and the first college of dentistry in the world have sprung. We can now have the satisfaction of contemplating the recent establishment of a journal of dental surgery in England, as a transatlantic approval of our course, and as lending the sanction of English surgery to our example. As a direct and natural consequence of the English journal, we anticipate the formation of an English association of dental surgeons, and, perhaps an English college of dentistry; and, still farther, may we not hope that the example of England may have its due effect upon the Continent, and that a journal, an association and college may also be established there—thus making a three-fold chain of defences against quackery—a triangular fortress to command the respect of the world, and a "three-fold cord not easily to be broken" of scientific associations, in different realms, of educated gentlemen, pledged to the improvement and protection of an art and science so preservative of health and beauty as is this branch of surgery! Such a three-fold intrenchment against erroneous practice would defend the civilized world against the inroads of quackery, and give an unexpected elevation to professional skill in this department unknown to those who have preceded us.

The establishment of a British national association of dental surgeons, now agitated in the metropolis of that empire, if achieved, we shall regard as a full-orbed omen of the accomplishment of our most ardent anticipations; and thenceforward we shall lay aside any fear for the final triumph of our art, and the elevation of its professors, *pari passu,* with those of general surgery, to all the high consideration which legitimately belongs to scientific gentlemen, engaged in remedying nature's defects and ameliorating human woes.

The insignia, or coat of arms of the American Society of Dental Surgeons, (also adopted by the British Journal,) designed and drawn by one of the most talented and highly esteemed members of this body, Dr. Maynard, of Washington City, is a polyandrian column, based upon a rock, surmounted by the lamp of science, throwing its rays above and around, encircled by the cognomen of the Association. I need not say that there was a happy genius in the conception; and should three such pillars rise from different empires of the globe, and should the radiance of each commingle with each, giving and receiving reciprocal splendour, communicating the warmth of friendship as well as the light of intellect, the effect would be as salutary to mankind as the scene would be morally sublime. It would of itself honour science, by adding strength and unity of purpose to its defences. Against three such columns, so planted on the rock of truth, and so illuminated by the "star-eyed" radiations of science, the waves of error might dash in anger, but they would dash in vain. In storm or calm, in "gloom or glory," those same changeless lights would shine, those same polyandrian columns stand unshaken, and those same adamantine rocks beat back the baffled surges, still bearing unmoved those glory-tipped pillars, as light-houses of scientific discovery, and as monuments of associated intellectual energy, devoted to the best interests of mankind.

Gentlemen of the Society—all our former meetings have been most emphatically re-unions for business—assemblies of true and zealous working men. Let not this character of promptitude, despatch and energy die away and subside, like some wave, driven upon a distant shore; but let it increase, gaining force and celerity to the end; ever making the Society, at each annual meeting, the great central beehive of the profession; or rather, let it be the overflowings of a vast central fountain, fed by springs that wander hither from every point of the compass, filling the fount to the brim—then rushing back, all over the country, in healthful, silvery streamlets, carrying comfort and happiness to the afflicted wherever they go—away, over hill and through rich valley, again to feed and replenish the springs that shall again flow back their wealth to the centre; or, let the members from the several states of the Union be like the caravans that annually journey to some metropolitan mart of splendour and opulence. Over hill and dale they move, and each setting sun sees them nearer their destined goal. They are laden with the harvest and products of a thousand different climes, and when, in the great exchange of nations, they have deposited their garnered stores, communicated and received the news of a hundred principalities, they return, laden with richer wealth, back to their native climes, carrying with them the treasures of India, and "the spices of Araby the blest;" going to the great convocation with the riches of a year's labour and experience, and coming back strengthened in the arduous path of duty, by all the knowledge, honour and encouragement that had accumulated at the centre.

The relation of this, the great central congress of this branch of science, to the state societies, (if this association shall determine on the formation of such,) when they shall have been established, will bear a resemblance to the stately and umbrageous banyan of the tropics—the parent stem towering heavenwards, spreading its arms wide on either hand, covered with broad and verdant foliage, and every limb that droops to the earth, borne down with a freight of beauty and fragrance, shall take root there, and become both branch and trunk—another brave and strong supporter to the flowering pile or tower of wild and enchanting beauty; and see! another and another of the branches, as they shall bend to become the family supporters, twin trunks of a multitudinous tree, pillars to a sylvan temple, wide enough to shelter a nation beneath its spreading arches; or as Milton says:

> "Branching so broad and long, that in the ground
> The bended twigs take root, and daughters grow
> About the mother tree, a pillar'd shade,
> High over-arched, and echoing walks between."

Gentlemen of the American Society of Dental Surgeons—I pause; not because my theme has become exhausted, or that the flame of my zeal in the cause of scientific dentistry flickers low on the altar where long it has burned. I only pause that your valued labours may begin—that the beehive of mental industry may begin to hum with your labour; that its stores may begin to swell with the wealth you have brought from your several fields of practice in distant cities and states; and that we may all become pleased and animated students once more, learning of each other in turn, and profiting by all.

Gentlemen, in this, the close of my salutatory, I welcome you to this anniversary meeting—to these halls of deliberation—to the labours of the session—to its enjoyments—to the greetings and hospitalities of the "Monumental City;" and may a gracious Providence preserve your lives many years, to achieve the greatest amount of good, both to yourselves and your fellow men.

